# BMP9 Promotes an Epithelial Phenotype and a Hepatocyte-like Gene Expression Profile in Adult Hepatic Progenitor Cells

**DOI:** 10.3390/cells11030365

**Published:** 2022-01-21

**Authors:** Annalisa Addante, Carlos González-Corralejo, Cesáreo Roncero, Nerea Lazcanoiturburu, Juan García-Sáez, Blanca Herrera, Aránzazu Sánchez

**Affiliations:** Department of Biochemistry and Molecular Biology, Faculty of Pharmacy, Health Research Institute of the Hospital Clínico San Carlos (IdISSC), Complutense University of Madrid (UCM), 28040 Madrid, Spain; annalisa.addante@gmail.com (A.A.); carlgo17@ucm.es (C.G.-C.); ceronce@ucm.es (C.R.); nerelazka@gmail.com (N.L.); juangarsaez@gmail.com (J.G.-S.)

**Keywords:** BMP9, oval cells, HGF, c-MET, differentiation

## Abstract

Bone morphogenetic protein 9 (BMP9), a member of the TGF-β superfamily, has emerged as a new player in chronic liver diseases (CLDs). Its levels increase in the fibrotic liver where it promotes fibrogenesis. It also regulates hepatic progenitor cells (oval cells in rodents), a cell population that contributes to repopulate the liver and recover functionality upon severe damage, but it can also be pro-fibrogenic, depending upon the hepatic microenvironment. Here we analyze the effect of chronic exposure to BMP9 in oval cells. We show that cells chronically treated with BMP9 (B9T-OC) display a more epithelial and hepatocyte-like phenotype while acquiring proliferative and survival advantages. Since our previous studies had revealed a functional crosstalk between BMP9 and the HGF/c-Met signaling pathways in oval cells, we analyzed a possible role for HGF/c-Met in BMP9-induced long-term effects. Data evidence that active c-Met signaling is necessary to obtain maximum effects in terms of BMP9-triggered hepatocytic differentiation potential, further supporting functionally relevant cooperation between these pathways. In conclusion, our work reveals a novel action of BMP9 in liver cells and helps elucidate the mechanisms that serve to increase oval cell regenerative potential, which could be therapeutically modulated in CLD.

## 1. Introduction

Bone morphogenetic proteins (BMPs), and specifically BMP9, have emerged as new regulators of liver physiology and pathology [1,2,3,4,5]. Recent studies are uncovering the function of BMP9 in different liver pathologic settings. Thus, BMP9 promotes proliferation, survival, invasion and cancer stem cell properties in hepatocellular carcinoma (HCC) cells, supporting a pro-tumorigenic role of BMP9 in the liver [6,7,8]. Additional in vitro and in vivo evidence reveal a role for BMP9 in the regulation of glucose and lipid metabolism as well as the hepatic inflammatory response, having been associated with non-alcoholic fatty liver disease (NAFLD) and non-alcoholic steatohepatitis (NASH) development, although somehow contradictory results have been described [9,10,11,12]. We and others have also demonstrated that BMP9 is a key regulator of liver fibrosis. Despite being transiently downregulated upon acute liver damage/repair, which may allow hepatocyte proliferation and plasticity, BMP9 is upregulated in experimental models of chronic liver fibrosis. More importantly, the loss of BMP9 signaling by different means (in BMP9-KO- and ALK1-Fc-treated mice, among other subjects) results in a significant decrease in the CCl_4_-induced fibrotic process [13,14]. Again, the controversy is heightened, as some studies have suggested a protective effect for BMP9 in liver fibrosis, and, to render an even more complicated picture, mouse strain-dependent responses have been found in terms of liver fibrosis upon genetic deletion of BMP9, which appear to be related to alterations in liver sinusoidal endothelial cell (LSEC) fenestrations and subsequent capillarization [1,2]. 

We have recently addressed the role of BMP9 in cholestatic disease by using a mouse model of cholestatic liver injury induced by a diet containing the porphyrinogenic compound 3,5 diethoxycarbonyl-1,4 dihydrocollidine (DDC). Our results show that the absence of BMP9 (BMP9-KO mice) results in reduced liver damage and an ameliorated fibrotic process, together with a greater ductular reaction and hepatic progenitor cell (HPC) expansion in response to DDC [15]. This, together with in vitro studies in oval cell lines showing BMP9-triggered cytotoxic effects, provided the first evidence of the role of BMP9 as a regulator of HPCs. 

HPCs (known as oval cells in rodents) constitute a bipotential cell population from the adult liver. In cases of chronic liver disease (CLD), when the regenerative capacity of the parenchymal cells is compromised, HPCs can be activated and contribute to sustain liver regeneration by expanding into liver parenchyma and differentiating into cholangiocytes and/or hepatocytes to compensate for the cellular loss, thus helping to maintain liver homeostasis [16,17]. However, some evidence supports a pro-fibrogenic role for these cells [18], which, together with the fact that they can also be targets of malignant conversion and become tumor-initiating cells [19], adds further confusion with respect to their as yet unclear role during liver injury. It has nevertheless been demonstrated that the fate of HPC/oval cells and their final contribution to the regenerative process is regulated by the hepatic stem/progenitor cell niche, a microenvironment composed of different cell types, including damaged hepatocytes and cholangiocytes, hepatic stellate cells (HSCs) and activated myofibroblasts, as well as the extracellular matrix (ECM) scaffold, growth factors, cytokines and other molecules released by niche cells [20,21]. One of these growth factors is BMP9, which is mainly produced by HSCs and upregulated upon their activation during liver fibrosis [13,14]. We have previously addressed the effects of acute treatment of HPC/oval cells with BMP9 [15,22]. Here we study how HPC/oval cells respond to chronic exposure to BMP9 as part of an in vitro approach to more closely mimic cell behavior in the context of a fibrotic liver. Furthermore, since we have uncovered a biologically relevant functional crosstalk between BMP9 and the HGF/c-Met signaling pathways in oval cells [22], we analyze if HGF/c-Met signaling has a role in BMP9-induced effects in this scenario. 

## 2. Materials and Methods

### 2.1. Cell Lines and Culture Conditions

Met^flx/flx^ and Met^−/−^ oval cell lines were generated and maintained as described [23]. To generate chronically BMP9-treated oval cells (B9T-OC), we adapted an established protocol to generate TβT-OC (OC chronically treated with TGF-β) [24]. Briefly, oval cells were treated for 4 days with a high concentration of BMP9 (5 ng/mL), which induces apoptosis [15]. Thereafter, the remaining cells were cultured in a medium supplemented with 10% fetal bovine serum (FBS) and a low concentration of BMP9 (1 ng/mL) until they reached confluency (passage 0, p0), considered the starting point for subculture. Cells were then submitted to subsequent passages when they reached confluency (always maintained in the presence of 1 ng/mL BMP9). After four passages it was considered a stable chronically treated cell line (B9T-OC, which stands for BMP9-treated oval cells). Two different B9T-OC lines were used for phenotypic and functional studies. Experiments were carried out in serum-free Dulbecco’s modified Eagle’s medium (DMEM, Sigma-Merck, Saint Louis, MO, USA). HepG2 human hepatocarcinoma cells were obtained from the European Collection of Cell Cultures (ECACC). Ras-oval cells were generated by lentiviral transduction of Met^flx/flx^ oval cells with oncogenic v-Ha-Ras followed by selection of GFP-positive cells by fluorescence-activated cell sorting, as previously described [24]. Both mouse recombinant HGF and human recombinant BMP9 were purchased from R&D Systems (Minneapolis, MN, USA)

### 2.2. Confocal Microscopy Analysis

To analyze protein expression by regular or confocal fluorescence microscopy, we used standard protocols previously described [23,25]. Cells were seeded on 2% gelatin-coated glass coverslips in DMEM supplemented with 10% FBS. For vimentin analysis, cells were fixed with 4% paraformaldehyde in PBS for 20 min at RT and incubated with a blocking solution containing 5% BSA and 0.3% Triton X-100 in PBS for 1 h at RT. Primary antibody incubation (Vimentin monoclonal antibody from Abcam (Cambridge, UK), Ab45939; 1:200 in 0.1% BSA-PBS) was performed for 15 h at 4 °C. Anti-mouse Alexa Fluor 488 conjugated secondary antibody (A11012, Invitrogen-Thermo Fisher Scientific, Waltham, MA, USA) was diluted 1:200 in PBS 1% BSA and applied for 1 h at RT. Antibody incubations were carried out in a humidity chamber to avoid evaporation. For visualization, cells were embedded in Vectashield (Vector Laboratories, Burlingame, CA, USA) mounting medium with DAPI (Sigma-Merck) and visualized using a Leica SP-8 fluorescence microscope. 

### 2.3. Analysis of Cell Number

Analysis of cell number was performed as described [15]. Cells were plated and serum starved for 2–4 h prior to treatment with different factors. At various time points, cells were harvested by trypsinization and viable cells were counted using trypan blue staining and a Neubauer chamber.

### 2.4. Measurement of Apoptotic Index 

Apoptotic index was measured as previously described [23] using a propidium iodide (PI) (Sigma-Merck) staining solution containing 5 μg/mL PI, 0.1% Triton X-100, 0.1 M EDTA and 25 U/mL RNAse (Sigma) (20 min incubation at 37 °C) in cells fixed with methanol:acetic acid (3:1) for 30 min at RT. Cells were washed and coverslipped using Mowiol mounting medium (Sigma). Cells undergoing apoptosis were scored under an inverted fluorescence microscope (Eclipse TE300, Nikon, Izasa Scientific, Alcobendas, Madrid, Spain) at high magnification (x60) following standard morphological criteria, that is, visualization of nuclear condensation, shrinkage and fragmentation. Quantification was performed in a blinded manner as follows: 10–20 microscopic fields per plate were randomly chosen throughout the entire plate in order to reach a minimum of 1000 cells per treatment. Both apoptotic and non-apoptotic nuclei were counted in each field to obtain the percentage of apoptosis/field. The score for each plate corresponded to the mean percentage of all fields. Each condition was run in triplicates, so the final score per condition was the mean percentage of three plates. 

### 2.5. Measurement of Caspase-3-like Enzymatic Activity

Caspase-3 activity was measured as previously described [22] by a fluorometric assay using Ac-DEVD-AMC as a fluorogenic caspase-3 substrate. Briefly, cells were lysed in 5 mM Tris-HCl (pH 8), 20 mM EDTA, 0.5% Triton X-100 for 30 min at 4 °C. Lysates were clarified by centrifugation at 13,000× *g* for 10 min. A reaction mixture containing 25 μL cell lysate, 325 μL assay buffer (20 mM HEPES pH 7.5, 10% glycerol, 2 mM dithiothreitol) and 20 μM caspase-3 substrate (BD Biosciences, Franklin Lakes, NJ, USA) was incubated for 2 h at 37 °C. Proteolysis of the synthetic substrate by active caspase-3 present in the lysates liberates the fluorogenic compound AMC, whose fluorescence was measured in a fluorimeter (fluorescence reader Infinite M200, TECAN, Männedorf, Sweden), Excitation/Emission, 380/440 nm). A unit of caspase activity is the amount of enzyme that will lead to a one-unit increase in the fluorescence intensity. Results are expressed as units of activity per microgram of protein.

### 2.6. RNA Isolation and Quantitative Reverse Transcriptase-Polymerase Chain Reaction

Total cellular RNA was isolated using the RNeasy Kit (Qiagen, Valencia, CA, USA). RNA yield and purity were analyzed using a spectrophotometer (ultraviolet–visible spectrophotometer Thermo Spectronic, Biomate 3, Rochester, NY, USA). Quantitative reverse transcriptase-polymerase chain reaction (RT-qPCR) was performed as described previously [26]. The primers used in the study are listed in the Supporting Information, Table S1.

### 2.7. Clonogenic Assay 

Colony-forming and self-renewal capacity was measured as previously described [24]. Briefly, 200 and 500 cells/well were seeded in a 6-well plate and allowed to grow into colonies in DMEM supplemented with 10% FBS. After 10 days, colonies were stained with crystal violet (0.2% solution in 2% ethanol) and the total number of colonies was quantified using the Image J program. 

### 2.8. Soft Agar Assays

Soft agar was performed as previously described [7]. Twenty thousand cells/well were seeded in 6-well plates and DMEM supplemented with 5% FBS and 0.45% agarose on the top of solidified agarose (0.9% in DMEM supplemented with 5% FBS). Three hundred μL DMEM per well were added twice weekly. Colonies were counted 3 weeks after seeding. Colonies with a diameter greater than 50 μm were scored.

### 2.9. Protein Isolation and Western Blot Analysis

Total protein extracts from cells were prepared in IP buffer (50 mM Tris pH 7.5; 150 mM NaCl; 1% NP40; 5 mM EGTA, 5 mM EDTA) supplemented with 1 mM phenylmethylsulfonyl fluoride, 10 μg/mL aprotinin and leupeptin, 1 mM sodium orthovanadate and 20 mM sodium fluoride. Western blotting procedures were carried out as previously described [23]. In 10–12% acrylamide sodium dodecyl sulfate–polyacrylamide electrophoresis gels, 30–80 μg of protein were separated and blotted to PVDF blotting membrane (Amersham, Chicago, IL, USA). Membranes were probed with the primary antibodies in Tris-buffered saline containing 0.1% Tween 20 and 0.5% non-fat dried milk or 0.5% bovine serum albumin, according to the manufacturer’s instructions. Detection was performed using the enhanced chemiluminescence (ECL) method in the gel documentation system Imager2 and Imager CHEMI Premium. Antibodies against the following proteins were used: albumin polyclonal antibody from Nordic Immunology (Susteren, The Netherlands) (RARa/Alb/PO); E-cadherin (610181) and N-cadherin (610921) monoclonal antibodies from BD Biosciences and β-actin (5-5441) mouse monoclonal anti-antibody from Sigma-Merck. Phospho-SMAD-2 (#3101), Phospho-SMAD1,5,8 (#9511 and #13820), Phospho-MET (#3077), Phospho-AKT (#9271) and Phospho-ERK (#9101) polyclonal antibodies from Cell Signaling Technology (Danver, MA, USA).

### 2.10. Analysis of Urea Production

Quantitative determination of urea concentration in the cell culture medium was performed using the SPINREACT kit (Spinreact, Girona, Spain), according to the manufacturer’s recommendation. Cells were seeded on 100 mm plates and the following day cells were left with a minimal amount of medium for 24 h. Then, cells were counted and media were collected. To perform the urea assay, 10 μL of cell media were transferred into a 1 cm cuvette with the working reagent, mixed and incubated for 10 min at RT. Then, sodium hypochlorite (NaClO) was added to each cuvette and mixed quickly. The reaction was incubated for 10 min at RT. Optical density (OD) at 580 nm was measured using a plate reader (Powerwave XS, Biotek, Winooski, VT, USA). The concentration of a standard urea sample containing 50 mg/dL urea was also measured. The urea concentration in the sample was calculated from the OD values:

mg/dL [Urea] = (OD Sample − OD Blank)/(OD Standard − OD Blank) × [Standard] mg/dL(1)

where OD Sample, OD Standard and OD Blank are OD580 nm values of the sample, standard and water blank, respectively. [Standard] is the concentration of the urea standard (50 mg/dL). The values obtained are expressed as μg urea/24 h/10^6^ cells. HepG2 cells were analyzed as a positive control.

### 2.11. Statistical Analysis

Statistical analysis was performed with paired Student’s *t*-test analysis or one-way ANOVA to calculate *p*-values once the normal distribution of data was verified using the Shapiro–Wilk test. A *p*-value ≤ 0.05 was considered statistically significant.

## 3. Results

### 3.1. Chronic Exposure to BMP9 Leads to Acquisition of Hepatocyte-like Properties in HPC/Oval Cells

**Figure 1:**
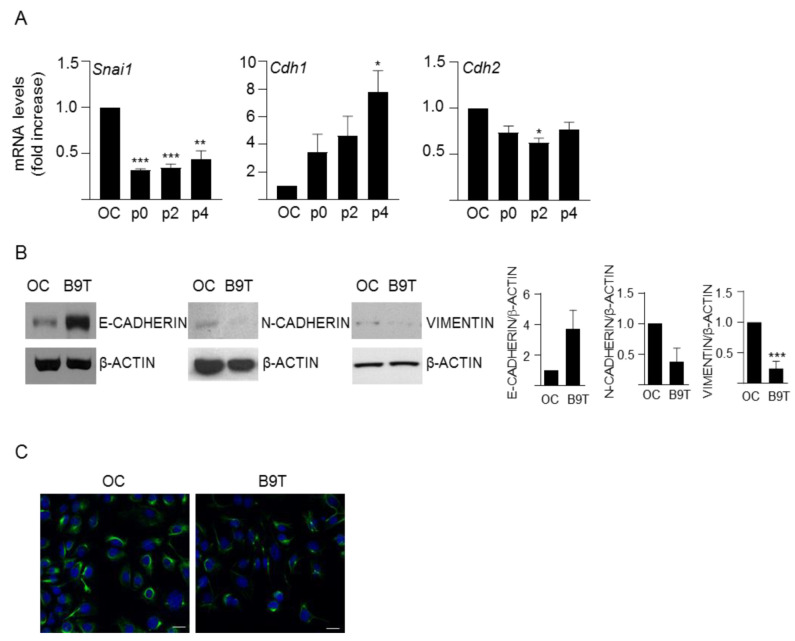
Analysis of EMT markers in B9T-OC. (**A**) RT-qPCR analysis for the expression of *Snai1*, *Cdh1* and *Cdh2* in oval cells (OC) and B9T-OCs at different passages (passage 0, 2 and 4). *Gusb* was used for normalization. Data are expressed relative to oval cells (assigned an arbitrary value of 1) and are mean ± SEM of three independent experiments. * *p* < 0.05, ** *p* < 0.01 and *** *p* < 0.001 B9T-OC vs. OC. (**B**) Western blot analysis of E-CADHERIN, N-CADHERIN and VIMENTIN in oval cells (OC) and established B9T-OCs (B9T). A representative experiment (left panel) and a densitometric analysis (right panel) are shown. Data corresponding to optical density values relative to loading control are mean ± SEM of three independent experiments and are expressed relative to OC samples (assigned an arbitrary value of 1). (**C**) Oval cells (OC) and B9T-OCs (B9T) were fixed and stained with the VIMENTIN antibody and an Alexa Fluor 488 conjugated secondary antibody. Nuclei were counterstained with DAPI. Representative confocal microscopy images from two to three experiments are shown. Scale bar = 20 μm.

**Figure 2:**
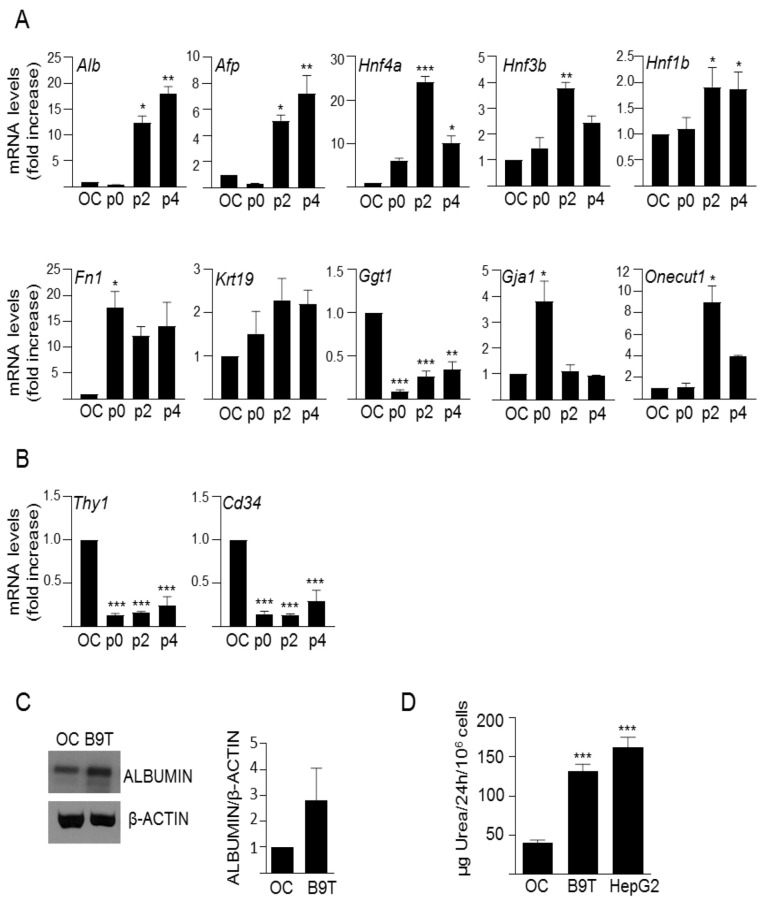
Analysis of lineage markers in B9T-OC cells. (**A**,**B**) RT-qPCR analysis for the expression of (**A**) hepatocyte and biliary markers, and (**B**) hematopoietic stem/progenitor cell markers. *Gusb* was used for normalization. Data are expressed relative to oval cells (assigned an arbitrary value of 1) and are mean ± SEM of three independent experiments. * *p* < 0.05, ** *p* < 0.01 and *** *p* < 0.001 B9T-OC vs. OC. (**C**) Western blot analysis for ALBUMIN and β-ACTIN (used as loading control) in oval cells (OC) and B9T-OCs (B9T) cultured in the absence of serum for 15 h. A representative experiment (left panel) and a densitometric analysis (right panel) are shown. Data corresponding to optical density values relative to loading control are mean ± SEM of three independent experiments and are expressed relative to OC samples (assigned an arbitrary value of 1). (**D**) Urea levels from oval cells (OC) and B9T-OCs supernatants were measured spectrophotometrically. HepG2 cells were used as positive control. Urea rate production was expressed as μg/24 h/10^6^ cells. Mean ± SEM (*n* = 5) is displayed. Statistical analysis was carried out using the paired *t*-test and data were compared to oval cells (OC), *** *p* < 0.001.

We adapted a protocol previously used to study chronic exposure to TGF-β [24,27] to chronically treat HPC/oval cells with BMP9 (Supplementary Figure S1A,B), as explained in the methods section. The cells were named B9T-OC, which stands for BMP9-treated oval cells. Once the B9T-OC were established, we performed a detailed phenotypic characterization. Since previous observations had shown that BMP9 is capable of inducing an epithelial-to-mesenchymal transition (EMT) process in liver cells [8], we first analyzed EMT markers, such as Snail (*Snai1*), one of the most relevant EMT-inducing transcription factors; E-cadherin (*Cdh1*), a cell–cell contact protein characteristic of epithelial cells; and N-cadherin (*Cdh2*) and Vimentin, two well-known mesenchymal markers. Interestingly, our results show that BMP9, rather than inducing an EMT process in HPC/oval cells, enhances the epithelial properties, as evidenced by increased expression levels of E-cadherin and decreased levels of Snail, N-cadherin and Vimentin (Figure 1). Next, we analyzed the expression of an array of lineage and phenotypic markers in comparison with the parental untreated oval cells (Figure 2A,B). We found that B9T-OC express higher levels of hepatocytic markers than parental oval cells, with a marked and sustained increase of albumin (*Alb*) and α-fetoprotein (*Afp*), two plasma proteins secreted by hepatocytes; as well as the hepatocyte nuclear factor 4α (*HNF4α*, *Hnf4a*); and fibronectin (*Fn1*), an ECM protein that promotes the differentiation of liver progenitor cells towards the hepatocyte phenotype [28]. More subtle were the changes in hepatocyte nuclear factor 3β (*HNF3*β, *Hnf3*β) and 1β (*HNF1*β, *Hnf1*β), but again, both of them were upregulated in B9T-OC. Biliary epithelial cell markers, such as cytokeratin 19 (CK19, *Krt19*) and gamma-glutamyltransferase 1 (GGT, *Ggt1*), were either not significantly regulated (CK19) or strongly downregulated (GGT) in B9T-OC. By contrast, hepatocyte nuclear factor 6 or Onecut (*Onecut1*) and Connexin 43 (*Gja1*) were sharply upregulated, but this regulation was transient, as by passage 4 mRNA expression levels had decreased to levels present in HPC/oval cells. Interestingly, hematopoietic stem cell markers, such as Thy1 (*Thy1*) and CD34 (*Cd34*), that were expressed in liver stem/progenitor cells are strongly downregulated in B9T-OC. Importantly, we confirmed that these changes in phenotypic and lineage markers were BMP9-dependent. For that, we performed the generation protocol with and without BMP9, that is, in a set of oval cells, BMP9 was not added in any step of the protocol. These cells were named NT, for non-treated, to distinguish them from the BMP9-treated cells (B9T-OC). The expression of *Cdh1*, *Alb*, *Afp*, *Hnf4a* and *Fn1* was much higher in B9T-OC than NT (Supplementary Figure S1C). Furthermore, as compared with NT cells, B9T-OC present an activation of the BMP9-triggered canonical pathway, determined by the Phospho-SMAD1,5,8 levels, in all stages of the generation process (Supplementary Figure S1D). As functional tests for HPC/oval cell differentiation toward a hepatocyte lineage, we analyzed albumin protein levels (Figure 2C) and urea production (Figure 2D), since they are among the most commonly used parameters to measure liver-specific functions in cells in vitro [29]. Albumin protein levels of established B9T-OC are notably higher than those of OC, in line with mRNA levels (Figure 2C). Regarding urea production, the amount of urea produced by parental oval cells was low, as expected for undifferentiated hepatic progenitor cells, but after BMP9 chronic treatment urea production was significantly increased, reaching levels similar to those found in HepG2 cells, a differentiated hepatic cell line (Figure 2D). These data provide evidence indicating that chronic exposure to BMP9 promotes the acquisition of hepatocyte-like specific functions in HPC/oval cells.

### 3.2. Chronic Exposure to BMP9 Confers Growth and Survival Advantages to HPC/Oval Cells

**Figure 3:**
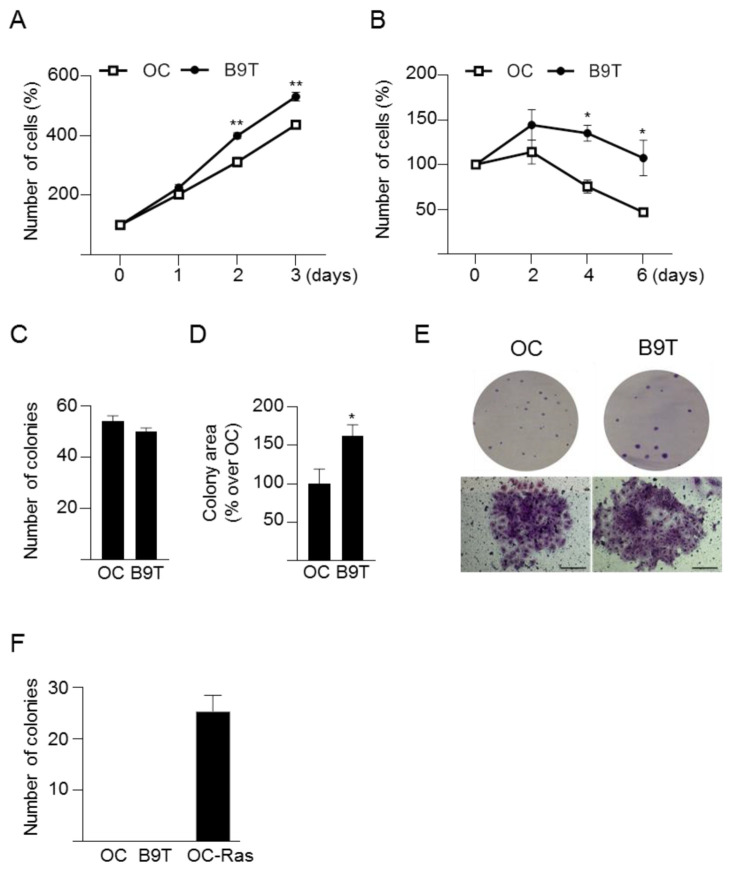
Analysis of B9T-OC growth capacity**.** (**A**,**B**). Oval cells (OC) and B9T-OC (B9T) were cultured in the presence (**A**) or absence (**B**) of 10% FBS for different periods of time and the number of cells was counted. Data are expressed with respect to day 0 (assigned as 100%) (mean ± SEM) and are from three to four independent experiments performed in triplicate. (**C**–**E**). Oval cells (OC) and B9T-OCs (B9T) were seeded at low density and maintained in 10% FBS-supplemented medium for up to 10 days. (**C**) Total number of colonies (mean ± SEM) (*n* = 6) and (**D**) the colonies area (*n* = 6) were determined. Colony area data are expressed relative to OC. (**E**) Representative images of clones from a plate (upper images) and representative phase contrast microscope images of individual foci (lower images, scale bar = 100 µm). (**F**) Soft agar assay with oval cells (OC), B9T-OC (B9T) and oval cells expressing v-Ha-Ras (OC-Ras). Colonies were counted after 3 weeks in culture (*n* = 4). * *p* < 0.05, ** *p* < 0.01, B9T-OC vs. OC.

**Figure 4:**
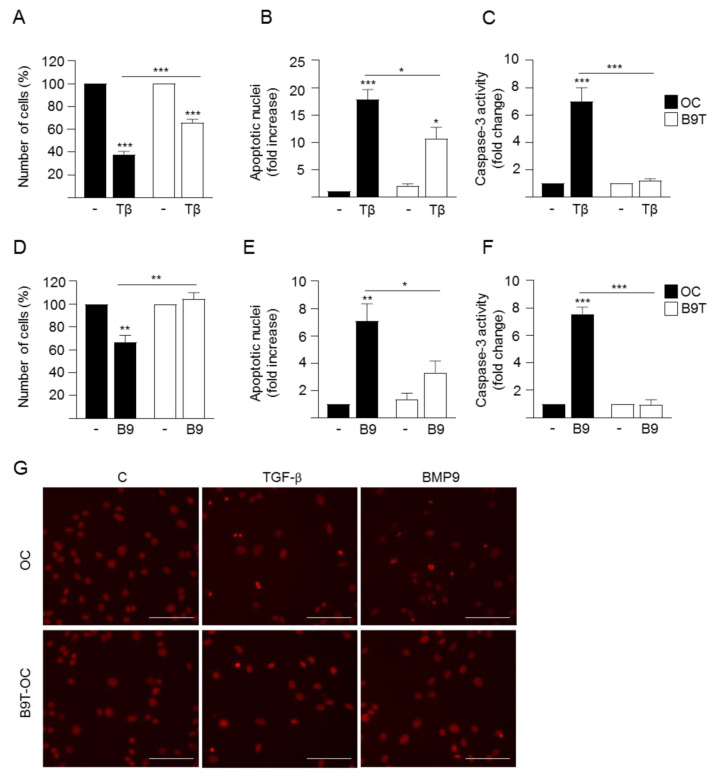
B9T-OC are more resistant to BMP9 and TGF-β-induced apoptosis than OC. (**A**–**C**). Oval cells (OC) and B9T-OC were serum-starved and incubated in the presence of TGF-β (2 ng/mL) for 48 h. (**A**) Cells were counted. Data from three independent experiments performed in triplicate are shown and expressed as percentage of untreated cells (mean ± SEM). (**B**) Apoptotic index was calculated by counting apoptotic nuclei after PI staining under a fluorescence microscope. A minimum of 1000 nuclei was counted per condition. Data from four independent experiments performed in triplicate (mean ± SEM) are shown and are expressed relative to untreated OC (assigned an arbitrary value of 1). (**C**) Caspase-3 activity. Data are mean ± SEM of four experiments and are expressed as a fold change of untreated cells. (**D**–**F**) OC and B9T-OC were serum-starved and incubated in the presence of BMP9 (2 ng/mL) for 48 h. (**D**) Cells were counted. Data from three independent experiments performed in triplicate are shown and expressed as percentage of untreated cells (mean ± SEM) (**E**). Apoptotic index was calculated as in (**B**). Data from four independent experiments performed in triplicate (mean ± SEM) are shown and are expressed relative to untreated OC (assigned an arbitrary value of 1). (**F**) Caspase-3 activity. Data are mean ± SEM of four experiments and are expressed as a fold change of untreated cells. (**G**) Representative images of propidium iodide staining taken under a fluorescence microscope used for quantification of apoptosis in (**B**,**E**). Scale bar = 50 μm. In all cases, data were compared with the untreated condition or as indicated, * *p* < 0.05, ** *p* < 0.01 and *** *p* < 0.001.

In order to deepen our understanding of the effects of chronic treatment with BMP9 on HPC/oval cell properties and behavior, we next evaluated the cell growth capacity in the absence or presence of serum. B9T-OC show higher growth rate in response to the mitogenic signals present in serum over their normal counterparts (Figure 3A). Even more interesting is the profile obtained in the absence of serum. While parental HPC/oval cells display a decrease in cell number, which is a consequence of the previously shown apoptotic response elicited by serum withdrawal [23], B9T-OC show an increase in cell number, suggesting an intrinsic greater capacity for proliferation and acquisition of apoptosis resistance (Figure 3B), which we confirmed by assaying caspase-3 activity in these cells (Supplementary Figure S2). We also performed clonogenic assays to test the clonal growth capacity of B9T-OC in comparison to untreated parental HPC/oval cells. Chronic treatment with BMP9 did not produce changes in the number of colonies, but the colony size was bigger in B9T-OC as compared to parental oval cells (Figure 3C–E). Importantly, a colony formation assay in soft agar was run using as a comparative reference HPC/oval cells transformed with oncogenic Ras (OC-Ras), and the results clearly show that B9T-OC did not acquire anchorage-independent cell growth capacities, indicating that chronic treatment with BMP9 does not drive malignant transformation (Figure 3F). Altogether, these data reveal that B9T-OC acquired growth advantages compared to parental HPC/oval cells but not malignant features. The results obtained in the absence of serum (Figure 3B and Figure S2) prompted us to analyze cell response to different cytostatic and/or pro-apoptotic stimuli. As we have already shown that TGF-β induces apoptotic cell death in HPC/oval cells [23], we treated cells with TGF-β and measured cell number, apoptotic index and caspase-3 activity. Results show that B9T-OC are more resistant to TGF-β-induced apoptosis (Figure 4A–C,G). We discarded an association between a decreased apoptotic response to TGF-β and alterations in TGF-β-triggered signaling in B9T-OC since no significant differences in the levels of phospho-SMAD2 were observed upon TGF-β treatment between B9T-OC and OC cell lines (Supplementary Figure S3A). An identical analysis was performed with acute treatment with BMP9, which we have demonstrated elicits an apoptotic response in oval cells [15]. Data show that BMP9 cytotoxic and apoptotic effects on oval cells are practically abolished in B9T-OC (Figure 4D–G), demonstrating that these cells are resistant to BMP9-suppressor effects, which again are not due to defects in BMP9 signaling, since no significant differences in the levels of phospho-SMAD1,5,8 upon acute BMP9 treatment were observed (Supplementary Figure S3B). Altogether, the data indicate that B9T-OC acquire resistance to pro-apoptotic stimuli.

### 3.3. c-Met Signaling Contributes to the Acquisition of B9T-OC Properties

**Figure 5:**
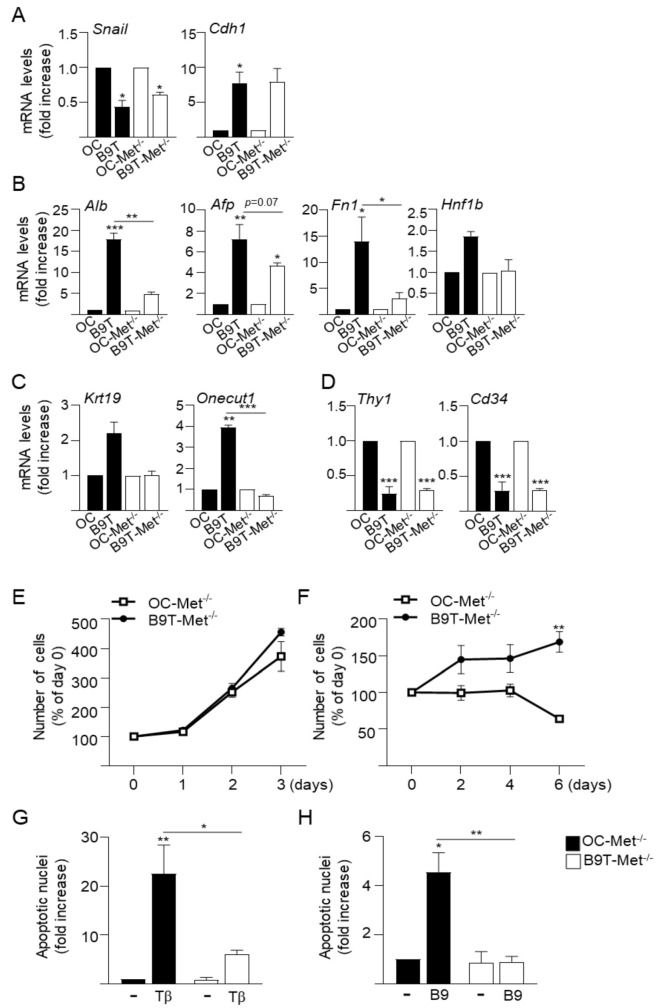
Analysis of the role of Met in the acquisition of B9T-OC properties. (**A**–**D**). RT-qPCR analysis for the expression of (**A**) EMT markers, (**B**) hepatocyte markers, (**C**) biliary cell markers and (**D**) hematopoietic stem/progenitor cell markers in OC, B9T-OC, OC-Met^–/–^ and B9T-Met^–/–^. *Gusb* was used for normalization. Data are expressed relative to OC and OC-Met^–/–^ (assigned an arbitrary value of 1) and are mean ± SEM of three independent experiments. (**E**,**F**). Met^–/–^oval cells (OC-Met^–/–^) and B9T-OC-Met^–/–^ cells (B9T-Met^–/–^) were cultured in the presence (**E**) or absence (**F**) of 10% FBS for different periods of time and the number of cells was counted. Data are expressed with respect to parental OC-Met^–/–^ (mean ± SEM) and are from three independent experiments performed in triplicate. Data were compared between cell lines. (**G**,**H**) Met^–/–^oval cells (OC-Met^–/–^) and B9T-OC-Met^–/–^ (B9T-Met^–/–^) were serum-starved and incubated in the presence of (**G**) TGF-β (2 ng/mL) or (**H**) BMP9 (2 ng/mL) for 48 h. Apoptotic index was calculated by counting apoptotic nuclei after PI staining under a fluorescence microscope. A minimum of 1000 nuclei was counted per condition. Data from three independent experiments performed in triplicate (mean ± SEM) are shown. Data were compared with the untreated condition or as indicated, * *p* < 0.05, ** *p* < 0.01 and *** *p* < 0.001.

Previous work from our laboratory has demonstrated the existence of a functional crosstalk between HGF/c-Met signaling and TGF-β [23,24,26] or BMP9 [22] that critically regulates oval cell phenotype and behavior. Therefore, we decided to study whether c-Met signaling contributes in some way to the phenotypic changes and functional advantages acquired by B9T-OC. Using the same approach described previously, we generated B9T-OC from c-Met mutant HPC/oval cells, oval cells expressing a non-functional c-Met receptor that lacks tyrosine kinase activity (Met^–/–^ oval cells, OC-Met^–/–^) generated in our lab [23]. The resultant cells were named B9T-OC-Met^–/–^ and were analyzed for the expression of phenotypic and lineage markers as done before with B9T-OC (Figure 5A–C). Similar to those seen in B9T-OC, B9T-OC-Met^–/–^ showed marked changes in gene expression profile with respect to parental OC-Met^–/–^. Regarding EMT markers, we did not observe differences in the regulation of Snail (*Snai1*), E-cadherin (*Cdh1*) or N-cadherin (*Cdh2*) in B9T-OC-Met^–/–^ as compared to regulation observed in B9T-OC (Figure 5A and Figure S4). Thus, in both cell lines Snail was downregulated and E-cadherin upregulated, meanwhile N-cadherin was not modulated in B9T-OC-Met^–/–^, while it was in B9T-OC, although differences between cell lines were not statistically significant. Interestingly, the modulation of some hepatocytic markers was attenuated in B9T-OC-Met^–/–^, as is the case for albumin, AFP and fibronectin, which were strongly upregulated in B9T-OC, but whose regulation in B9T-OC-Met^–/–^ was significantly diminished or lost. Furthermore, *HNF-1β* and biliary epithelial cell markers that were modulated in B9T-OC, such as Onecut and CK19, were not modulated in B9T-OC-Met^–/–^, and no differences were seen in *HNF4a* or *HNF3β*. Downregulation of the hematopoietic markers Thy1 and CD34 was similarly observed in both cell lines (B9T-OC and B9T-OC-Met^–/–^) (Figure 5B–D and Figure S4). As a complementary approach to further confirm the involvement of c-Met in the regulation of lineage markers in B9T-OC, we chemically mimicked B9T-OC-Met^–/–^ by treating B9T-OC with the Met inhibitor PHA665752, an ATP competitive c-Met inhibitor. The efficacy of PHA665752 to inhibit HGF-induced activation of the c-Met receptor and its downstream targets ERK-MAPKs and AKT was demonstrated in B9T-OC (Supplementary Figure S5A). Importantly, treatment of B9T-OC with PHA665752 led to a decrease in the expression levels of some of the lineage markers upregulated in B9T-OC results consistent with those obtained for B9T-OC-Met^–/–^ (Supplementary Figure S5B) and that altogether support a role for c-Met signaling in a lineage-specific shift triggered by BMP9 in oval cells. However, the lack of c-Met catalytic activity did not affect the growth and survival properties of B9T-OC, so that B9T-Met^–/–^ showed similar behavior to B9T-OC both in terms of growth rate and apoptotic resistance (Figure 5E,F vs. Figure 3A,B and Figure 5G,H vs. Figure 4).

## 4. Discussion

Although the role of BMP9 in liver fibrosis and chronic injury is far from being fully characterized, with results pointing in opposite directions [1,2], we and others have provided evidence that BMP9 can behave as a profibrotic factor in the liver [13,14]. Here, we attempted to analyze HPC/oval cells as a target of BMP9 in the fibrotic liver, for which we established an in vitro approach based on chronic exposure of HPC/oval cells to BMP9, a context somehow mimicking the in vivo situation. We reasoned that a detailed phenotypic and functional characterization of this cell population (B9T-OC) would enable us to predict the impact of the regulatory effect of BMP9 on the fate of these cells in the context of chronic liver damage and to better understand their potential relationship with liver fibrosis development and progression.

Strikingly, gene expression analysis of phenotypic markers revealed that long-term exposure to BMP9 leads to a decrease in Snail, the master regulator and inducer of EMT [30], together with an increase in the epithelial marker E-cadherin and a decrease in mesenchymal markers, N-cadherin and Vimentin (Figure 1). These results pointed to an acquisition of a more epithelial, rather than mesenchymal phenotype, as opposed to previous work in HCC cells where BMP9 promoted an EMT [8]. Furthermore, based on the expression profile of hepatic and stem/progenitor cell markers in HPC/oval cells and B9T-OC, BMP9 seems to promote a step-forward in the hepatic differentiation process of mouse oval cells toward hepatocytes. This is supported by our data showing that B9T-OC expressed higher levels of hepatocyte markers, albumin and AFP, along with transcription factors known to be critical during hepatocyte differentiation, including HNF3β, an early hepatocyte differentiation marker [31]; HNF1β, which has been reported to have a crucial role in hepatogenesis in vitro, promoting expression of several hepatic lineage-specific markers and functional properties [32]; and HNF4α, which is known to promote the terminal differentiation of hepatocytes [31]. Fibronectin upregulation is also likely related to promotion of the differentiation towards a hepatocyte-like cell, as seen before [28]. It is worth mentioning the profound downregulation of CD34 and Thy-1 expression in B9T-OC, which are hematopoietic markers typically expressed in stem/progenitor cells and cancer stem cells, also expressed in both fetal and adult liver progenitors and whose expression decreases as the cells differentiate into cholangiocytes or hepatocytes [33,34,35]. Ultimately, we have shown that B9T-OC acquire urea production capacity, a specific function of mature hepatocytes [29]. These data are in agreement with our previous data showing that BMP9 counteracts the culture-provoked EMT in mouse hepatocytes and enhances the expression of metabolic enzymes, while BMP9KO hepatocytes express higher levels of mesenchymal markers (such as vimentin) and lower levels of epithelial markers (E-cadherin) and hepatocytic lineage markers (such as albumin and HNF4α) [13]. Altogether, our work highlights the versatile actions of BMP9 in some way related to the malignant or non-malignant cell phenotype, so that in HCC cells BMP9 induces EMT [8], whereas in normal hepatocytes and HPC/oval cells BMP9 stabilizes and promotes a more epithelial and mature phenotype. The mechanisms behind such versatility and complexity are far from being understood and will require intense future research. However, in this regard, we provide clear evidence pointing to the establishment of interesting crosstalk between BMP9 signaling and other signaling pathways as one potential mechanism able to modulate cell responses. In fact, here we show that c-Met activity is necessary to obtain maximum effects of long-term BMP9 treatment in terms of hepatocytic differentiation potential, as genetic or pharmacological inhibition of c-Met negatively affects the gene expression shift, significantly reducing the upregulation of hepatocytic markers (Figure 5). These data add to our previous studies, demonstrating a functional interplay between BMP9 and the HGF/c-Met signaling pathways that critically regulates HPC/oval cell survival [22]. Our new data indicate that HGF/c-Met-BMP9 crosstalk also impacts on other essential biological processes, such as cell differentiation. HGF/c-Met involvement in the control of hepatic lineage differentiation is not really surprising since it has been known for years that this pathway is essential for liver development [34,36,37]. HGF is an important component of the growth factor/hormone cocktails used to drive hepatocyte generation from pluripotent stem cells (iPSCs and ESCs) [38]. Furthermore, Kitade et al. have elegantly demonstrated that c-Met signaling is a strong inducer of hepatocyte differentiation in HPC/oval cells via activation of AKT and STAT3 [39]. In any event, the importance of the HGF/c-Met axis in the regulation of HPC/oval cells is remarkably broad and encompasses differentiation, growth, survival, migration, invasion and morphogenesis, as we and others have shown [23,25], and is required for the repopulating capacity of these cells [40]—a fact that is, in the end, illustrated by a complete abolishment of the oval cell-mediated regenerative response in the absence of c-Met in the liver [41]. How BMP9 and c-Met mechanistically interact to control HPC/oval cell differentiation is unclear and needs to be addressed in future studies.

It is somehow surprising that while BMP9 promotes a more mature and epithelial phenotype in HPC/oval cells, at the same time it confers clear advantages in cell proliferation and survival (Figure 3 and Figure 4), as this could be easily associated with a more aggressive phenotype. It is interesting to highlight that these traits do not depend on Met activity (Figure 5), in spite of its known mitogenic and survival effects in HPC/oval cells, indicating that B9T-OC activated alternative pathways to sustain these processes. The elucidation of such pathways is a pending issue, but, based on our previous data, we could hypothesize that an alteration in the balance of activation of anti-apoptotic SMAD1 and pro-apoptotic p38MAPK pathways controlling oval cell death/survival upon BMP9 treatment [22] might be determinant. Additionally, since TGF-β-induced apoptosis in HPC/oval cells is an oxidative stress-dependent process [26], it would be interesting to test if B9T-OC display a potentiation of antioxidant defenses that protects them against TGF-β. In any event, the fact that B9T-OC are resistant to TGF-β-triggered cell death could be considered an advantageous feature in the context of the fibrotic liver, where this cytokine is greatly expressed [42,43], and could have an influence on the balance between its suppressor and protumorigenic signaling arms, but this is something that will have to be examined in the future. Pending resolution of the mechanistic aspects/details, we have shown that regardless of their enhanced proliferative and survival capacities, B9T-OC do not form colonies in soft agar (Figure 3), proving that long-term exposure to BMP9 by itself is not sufficient to promote the malignant transformation of HPC/oval cells. The strong downregulation of Thy-1 in B9T-OC can also be seen as a trait opposite to a malignant phenotype, as Thy-1 is often used as a biomarker of several tumors, including HCC and cancer stem cells [44,45]. All in all, our data show that long-term exposure of HPC/oval cells to BMP9 promotes the acquisition of a more differentiated, proliferative and apoptosis-resistant, but nonetheless non-malignant phenotype, which would certainly modulate the regenerative potential of HPC/oval cells.

In conclusion, our work shows for the first time that in a context of chronic exposure to BMP9, such as that of a fibrotic liver, HPC/oval cells can be pushed to differentiate into hepatocytes while acquiring still unknown mechanisms that promote their proliferation and survival. These cellular features do not seem to fit with a pro-fibrotic role, but rather with an enhanced and/or accelerated regenerative potential of the cells, consequently improving the liver’s regenerative response and facilitating the restoration of liver function upon injury. We also show that active c-Met signaling is necessary to obtain maximum hepatocytic differentiation potential, further evidencing the relevance of a functional cooperation between these two pathways. While featuring the complexity of the actions of BMP9 in liver cells, actions that appear to depend on the status of the cells and likely the stage of the disease, our work helps to elucidate the mechanisms contributing to an increase in OC regenerative potential that could be therapeutically modulated in CLD.

## Data Availability

Not applicable.

## Supplementary Materials

The following supporting information can be downloaded at: https://www.mdpi.com/article/10.3390/cells11030365/s1, Table S1: Primer sequences used in quantitative PCR; Figure S1: Generation of BMP9-treated oval cells (B9T-OC); Figure S2: Smad2 and Smad1,5,8 phosphorylation in response to TGF-β and BMP9, respectively, in B9T-OC; Figure S3: Analysis of caspase-3 activity in serum starved OC and B9T-OC; Figure S4: Comparison of phenotypic and lineage markers expression in B9T and B9T-Met^–/–^ OC; Figure S5: Effect of Met inhibitor on B9T-OC gene expression profile.
