# Supplementary material for: BMP9 Promotes an Epithelial Phenotype and a Hepatocyte-like Gene Expression Profile in Adult Hepatic Progenitor Cells
Source: Cells. 2022 Jan 21;11(3):365. doi: 10.3390/cells11030365 (PMC8834621; doi:10.3390/cells11030365)
Supplement: Supplementary file 1 [file cells-11-00365-s001.zip › cells-1411892-supplementary.pdf]

Supplementary table S1

Protein	Gene (*Mus musculus*)	Forward primer (5′-3′)	Reverse primer (5′-3′)
AFP	*Afp*	TGTTGCCAAGGAAACTCG	GCAGCACTCTGCTATTTTGC
E-CADHERIN	*Cdh1*	CAGCCTTCTTTTCGGAAGACT	GGTAGACAGCTCCCTATGACTG
GUSB	*Gusb*	AAAATGGAGTGCGTGTTGGGTCG	CCACAGTCCGTCCAGCGCCTT
HNF3B	*Foxa2 Hnf3b*	ACTGGAGCAGCTACTACG	CCCACATAGGATGACATG
GGT	*Ggt1*	TGCGGTTTCAGAGGATGGCAG	AACAGGATGCCACTGACCCGA
HNF1B	*Hnf1b*	TCTCAGAACCTCATCAGACC	GCTAGCCACACTGTTAATGACC
HNF4A	*Hnf4a*	GGCATGGATATGGCCGACTAC	TTCAGATGGGGACGTGTCATT
HNF6	*Onecut1*	CCTGGAGCAAACTCAAGTCG	GTCCTTCCCGTGTTCTTGC
Ck19	*Krt19*	GTGCCACCATTGACAACTCC	AATCCACCTCCACACTGACC
SNAIL	*Snai1*	TCCAAACCCACTCGGATGTGAAGA	TTGGTGCTTGTGGAGCAAGGACAT
ALBUMIN	*Alb*	ACGTGTGTTGCCGATGAGTCTGC	CAGCAGTCAGCCAGTTCACCAT
CD34	*Cd34*	AGCCACCAGAGCTATTCCCGA	GTTCCAGCTCCAGCTTTCTCC
THY	*Thy*	CCACCCCTGGTGAAAACTGC	GGGTTCATGGTGCAAGAGT
N-CADHERIN	*Cdh2*	ACCTGAGAGTGAACGGAGACCC	TGCATGGAGAACTCGGGTGCCT
FIBRONECTIN	*Fn1*	ATGTGGACCCCTCCTGATAGT	GCCCAGTGATTTCAGCAAAGG
CONNEXIN43	*Cx43 CjA1*	ATGCTACGACCACCACTTCC	AGGCCACATGCATAGCTACC

Supplementary table S1. Primer sequences used in quantitative reverse transcriptase-polymerase chain reaction (RT-qPCR)

Supplementary Figure S1

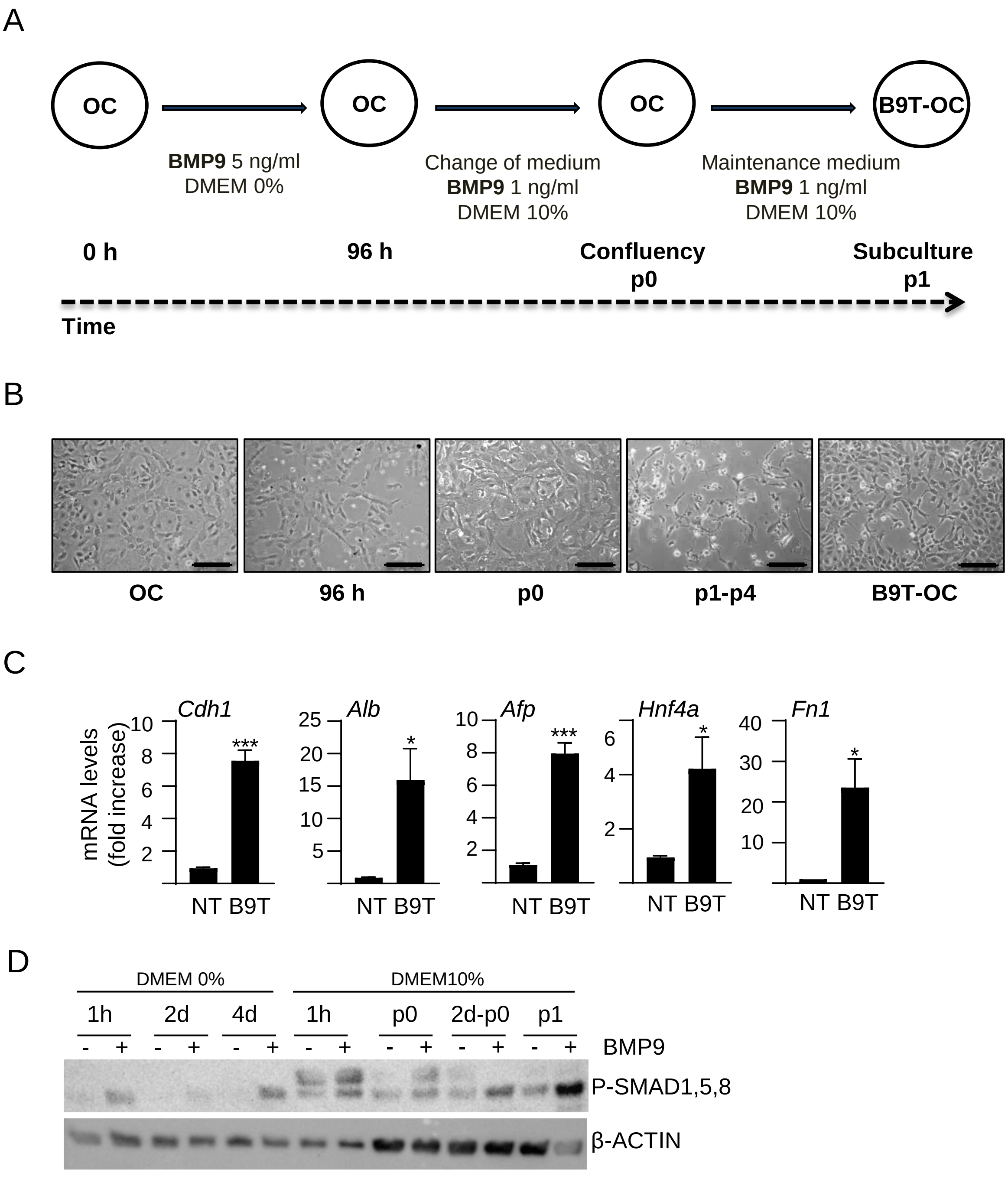

Supplementary figure S1. Generation of BMP9-treated oval cells (B9T-OC).
A. Schematic representation of the protocol used for the generation of B9T-OCs. **B.** Contrast microscopy images of the cells during different phases of the B9T-OC generation process. Scale bar= 100 µm. **C**. OC were submitted to the generation protocol with (B9T) or without BMP9 (NT) and mRNA was isolated. RT-qPCR analysis for the expression of *Cdh1,Alb, Afp, Hnf4a and Fn1* was performed. *Gusb* was used for normalization. Data are expressed relative to NT (assigned an arbitrary value of 1) and are mean ± S.E.M of 3 independent experiments. **D**. OC were submitted to the generation protocol with or without BMP9. At different time points, protein was isolated and western blot for the analysis of Phospho-SMAD1 was performed. β-ACTIN was analyzed as loading control. (2d-p0, stands for cells 2 days after p0). **p*<0.05 , ***p*<0.01, ****p*<0.001.

Supplementary Figure S2

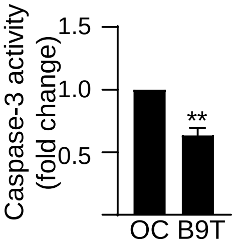

Supplementary figure S2. Analysis of caspase-3 activity in serum starved OC and B9T-OC.

OC and B9T-OC were serum starved for 4 days and caspase-3 activity was determined. Data are expressed relative to OC cells (assigned an arbitrary value of 1) and are from 3 independent experiments performed in triplicate (mean ± S.E.M.). **p<0.01.

Supplementary Figure S3

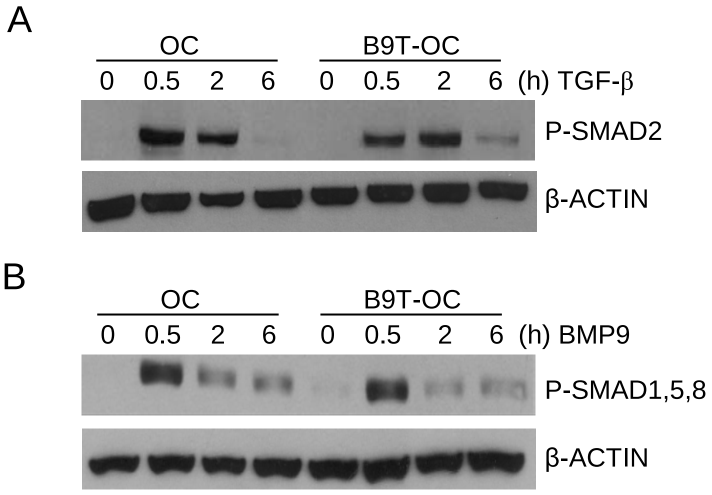

Supplementary figure S3. SMAD2 and SMAD1,5,8 phosphorylation in response to TGF-β and BMP9, respectively, in B9T-OC.

A-B. Oval cells (OC) and B9T-OC were maintained in the absence of serum for 16 h and then stimulated with TGF-β (2 ng/ml) (**A**) or BMP9 (2 ng/ml) (**B**) for 0.5, 2h and 6h. A representative western blot image from two independent experiments is shown. β-ACTIN was analyzed as loading control.

Supplementary Figure S4

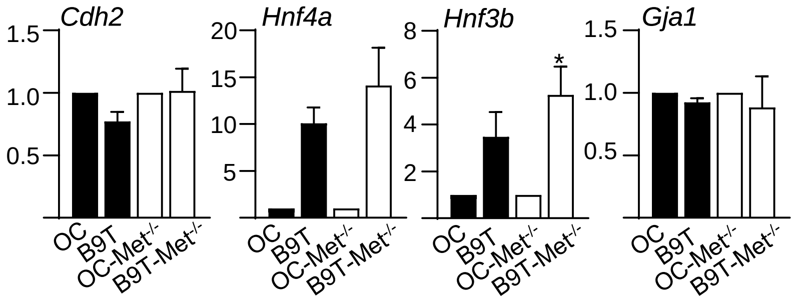

Supplementary figure S4. Comparison of phenotypic and lineage markers expression in B9T and B9T-Met$^{-/-}$ OC.

RT-qPCR analysis for the expression of *Cdh2, Hnf4a, Hnf3b* and *Gja1* in OC, B9T-OC, OC-Met$^{-/-}$ and B9T-Met$^{-/-}$. *Gusb* was used for normalization. Data are expressed relative to OC and OC-Met$^{-/-}$ (assigned an arbitrary value of 1) and are mean ± S.E.M of 3 independent experiments. *p<0.05.

Supplementary Figure S5

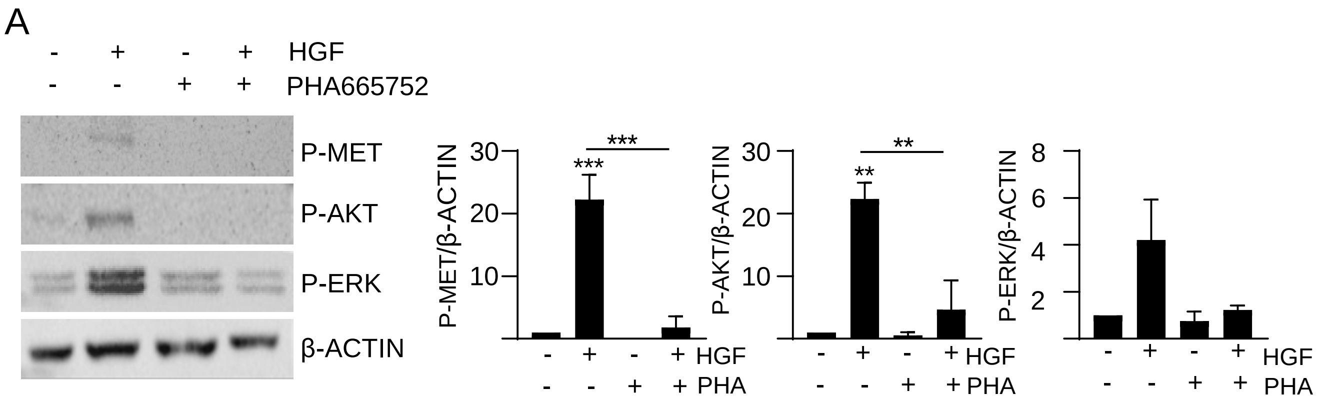

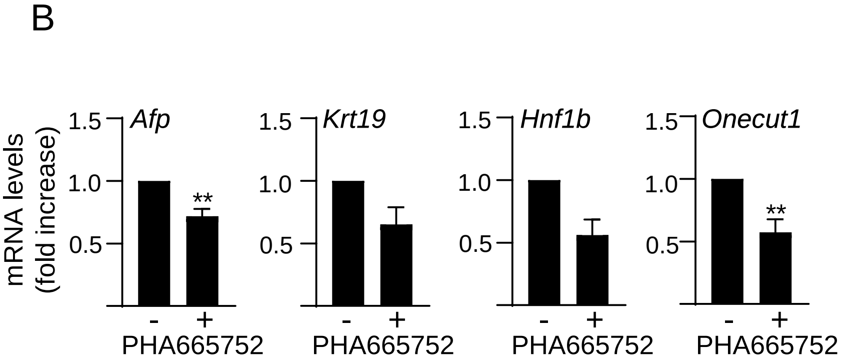

Supplementary figure S5. Effect of Met inhibitor on B9T-OC gene expression profile.
A. B9T-OC were serum starved, pre-treated with Met inhibitor PHA665752 (5 μM) for 1 h and treated with HGF (40 ng/ml) for 10 min. Total proteins were isolated and western blot assay was performed for the analysis of the indicated proteins using β-ACTIN as loading control. A representative experiment (left panel) and a densitometric analysis (right panel) are shown. Data corresponding to optical density values relative to loading control are mean ± S.E.M of 3 independent experiments and are expressed relative to untreated samples (assigned an arbitrary value of 1). **B.** RT-qPCR analysis for the expression of hepatocyte and biliary cell markers. *Gusb* was used for normalization. Data are expressed relative to B9T-OC (assigned an arbitrary value of 1) and are mean ± S.E.M of 3 independent experiments. $^{**}p<0.01$, $^{***}p<0.001$.
